# Use of the 988 Suicide and Crisis Lifeline at National, Regional, and State Levels

**DOI:** 10.1001/jamanetworkopen.2025.14323

**Published:** 2025-06-09

**Authors:** Jonathan Purtle, Amanda I. Mauri, Sachini Bandara, Elizabeth A. Stuart

**Affiliations:** 1 Department of Public Health Policy & Management, New York University School of Global Public Health, New York, New York; 2 Department of Mental Health, Johns Hopkins Bloomberg School of Public Health, Baltimore, Maryland; 3 Department of Biostatistics, Johns Hopkins Bloomberg School of Public Health, Baltimore, Maryland

## Abstract

This cross-sectional study describes incidence and prevalence of use of the 988 Suicide and Crisis Lifeline from its launch in July 2022 to December 2024.

## Introduction

The 988 Suicide and Crisis Lifeline launched nationally in July 2022. Little prior work has quantified use of 988,^1,2,3^ especially at subnational levels. In this study, we calculated lifetime and past-year incidence rates of 988 contacts and estimated prevalence of 988 use at national, regional, and state-levels.

## Methods

This cross-sectional study was approved by the New York University institutional review board with a waiver of informed consent because we did not have access to any individual-level data and the study was not considered human participants research. Results are reported in accordance with the STROBE reporting guideline for cross-sectional studies.

Comprehensive, monthly, state-level data on 988 contact volume across all modalities (ie, call, text, and chat) for the 30 months between July 2022 (when 988 launched) and December 2024 were obtained from Vibrant Emotional Health. Demographic information about 988 contacts is not available, nor is information about repeat contacts. July 2023 and July 2024 population size estimates were obtained from the US Census.

All analyses were conducted at national, Census region, and state levels. First, we calculated lifetime and past-year (2024) incidence rates of 988 contacts per 1000 population. Second, we estimated lifetime and past-year 988 use prevalence by dividing the number of 988 contacts by the size of the 2023 and 2024 populations, respectively. We then adjusted these prevalence estimates to reflect assumptions about repeat contacts to 988. Using data on repeat contacts to the Crisis Text Line to inform our assumptions, we adjusted the lifetime prevalence estimate to reflect the assumption that every person who contacted 988 used it a mean of 2.0 times and adjusted the past-year prevalence estimate to reflect the assumption that every person who contacted 988 used it a mean of 1.5 times. Choropleth maps were created to show state-level variation in 988 use. Analyses were conducted using SPSS statistical software version 30 (IBM) from March 1 to 31, 2025.

## Results

Nationally, 988 was contacted 16 333 707 times between July 1, 2022, and December 31, 2024, with 1 792 123 contacts (11.0%) rerouted to the Veterans Crisis Line. Of all contacts, 11 453 863 (70.1%) were calls, 2 942 852 (18.0%) were texts, and 1 936 992 (11.9%) were chats.

In this 30-month period, the national lifetime 988 contact incidence rate was 48.9 per 1000 population, and the estimated lifetime 988 use prevalence was 2.4% (Table). The national past-year 988 contact incidence rate was 23.7 per 1000 population, and the estimated past-year 988 use prevalence was 1.6%.

**Table.** Contacts to the 988 Suicide and Crisis Lifeline, July 2022 to December 2024^a^

Region	988 Contacts, No.		988 Contact incidence rate per 1000 population		Repeat contact–adjusted 988 use prevalence estimate, %	
	Total (lifetime)	2024 (Past year)	Total (lifetime)	2024 (Past year)	Lifetime (lifetime)	2024 (Past year)
Overall US	16 333 707	8 050 380	48.9	23.7	2.4	1.6
Census region						
West	4 445 070	2 167 638	56.3	27.1	2.8	1.8
Northeast	3 017 363	1 557 574	53.0	26.9	2.6	1.8
Midwest	3 508 011	1 681 945	50.9	24.2	2.5	1.6
South	5 363 263	2 643 223	41.4	20.0	2.1	1.3

^a^ Contacts include all calls, texts, and chats routed to the 988 Suicide and Crisis Lifeline and are inclusive of contacts that are subsequently rerouted to the Veterans Crisis Line, LGBTQ+ Line, Native Line, or national backup center. For calls and chats, contacts were assigned to states according to the first 6 digits of the contactor’s phone number. Texts were assigned to states according to the zip code the chatter enters during a prechat survey. National implementation of place-based 988 georouting began in November 2024. For lifetime estimates (July 2022 to December 2024), 2023 population data were used.

Regionally, the past-year 988 contact incidence rate was highest in the West and lowest in the South (27.1 vs 20.0 per 1000 population) and the same was true for the past-year estimate of 988 use prevalence (1.8% vs 1.3%). At the state-level, past-year 988 contact incidence rates ranged from highs of 45.3 and 40.2 per 1000 population in Alaska and Vermont, respectively, to lows of 12.5 and 14.4 per 1000 population in Delaware and Alabama, respectively (Figure).

**Figure.**
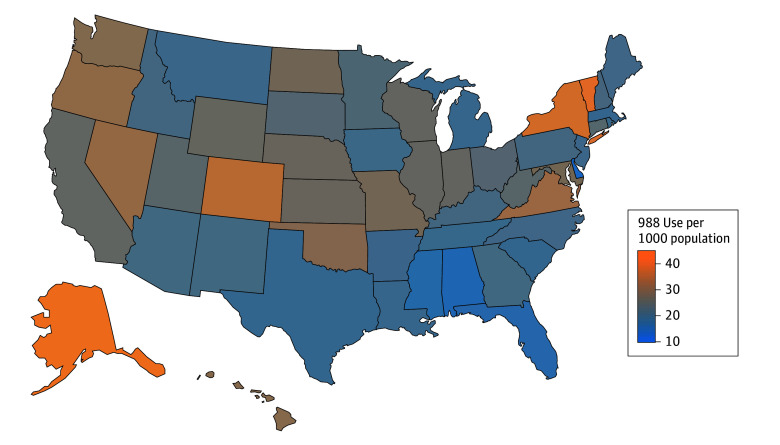
State Variation in Contacts to the 988 Suicide and Crisis Lifeline, January 2024 Through December 2024 Contacts include all calls, texts, and chats routed to the 988 Suicide and Crisis Lifeline and are inclusive of contacts that are subsequently rerouted to the Veterans Crisis Line, LGBTQ+ Line, Native Line, or national backup center. For calls and chats, contacts were assigned to states according to the first 6 digits of the contactor’s phone number. Texts were assigned to states according to the zip code the chatter enters during a prechat survey. National implementation of place-based 988 georouting began in November 2024. Rates are based on 2024 population data.

## Discussion

The findings of this cross-sectional study suggest that although 988 has been contacted more than 16 million times since its launch, there remains opportunity to increase 988 use. For example, the past-year 988 contact rate of 23.7 per 1000 is less than half that of the rate of adult emergency department visits that include a mental health diagnosis (53.0 per 1000 population).^4^ Furthermore, there was regional and state-level variation in 988 use. This is consistent with state variation in funding for and legislative attention toward 988,^2,5^ both of which may affect awareness and use. Lower rates of 988 use in the South, which is more politically conservative than other regions, is also consistent with prior research showing less favorable attitudes toward 988 among Republicans than Democrats.^6^

Study limitations relate to 988 contacts being assigned to states according to area code, uncertainty about the 988 repeat contact rates, and state-level variation in the existence of other crisis lines that are not part of the 988 network. Despite these inherent data limitations, our findings suggest that there are opportunities to increase 988 use, especially in southern states.

## Notes

Supplement.
Data Sharing Statement

## References

[1] National Alliance of Mental Illness. Poll of public perspectives on 988 & crisis response (2024). Accessed May 1, 2025. https://www.nami.org/support-education/publications-reports/survey-reports/poll-of-public-perspectives-on-988-crisis-response-2024/

[2] Purtle J, Chance Ortego J, Bandara S, Goldstein A, Pantalone J, Goldman ML. Implementation of the 988 Suicide & Crisis Lifeline: estimating state-level increases in call demand costs and financing. J Ment Health Policy Econ. 2023;26(2):85-95. 37357873 PMC10758993

[3] Purtle J, McSorley AMM, Adera AL, Lindsey MA. Use, potential use, and awareness of the 988 suicide and crisis lifeline by level of psychological distress. JAMA Netw Open. 2023;6(10):e2341383. 37906197 10.1001/jamanetworkopen.2023.41383 PMC10618841

[4] Peters ZJ, Santo L, Davis D, DeFrances CJ. Emergency Department Visits Related to Mental Health Disorders Among Adults, by Race and Hispanic Ethnicity: United States, 2018-2020. National Center for Health Statistics; 2023. doi:10.15620/cdc:123507 36939656

[5] Purtle J, Soltero M, Crane ME, McSorley AMM, Knapp M, Drapeau CW. State legislator social media posts about the 988 suicide and crisis lifeline. JAMA Netw Open. 2023;6(10):e2339845. doi:10.1001/jamanetworkopen.2023.39845 37883089 PMC10603493

[6] Callaghan T, Ferdinand AO, Motta M, Lockman A, Shrestha A, Trujillo KL. Public attitudes, inequities, and polarization in the launch of the 988 lifeline. J Health Polit Policy Law. 2024;49(3):473-493. doi:10.1215/03616878-11066312 37987198

