# Supplementary material for: Use of the 988 Suicide and Crisis Lifeline at National, Regional, and State Levels
Source: JAMA Netw Open. 2025 Jun 9;8(6):e2514323. doi: 10.1001/jamanetworkopen.2025.14323 (PMC12150192; doi:10.1001/jamanetworkopen.2025.14323)
Supplement: Supplement. — Data Sharing Statement [file jamanetwopen-e2514323-s001.pdf]

Data Sharing Statement

Purtle. Use of the 988 Suicide and Crisis Lifeline at National, Regional, and State Levels.
JAMA Netw Open. Published June 09, 2025. doi:10.1001/jamanetworkopen.2025.14323

Data
Data available: Yes
Data types: Deidentified participant data
How to access data: From author
When available: With publication

Supporting Documents
Document types: None

Additional Information
Who can access the data: Researchers
Types of analyses: Any purpose
Mechanisms of data availability: signed data access agreement
